# Next generation sequencing as a novel tool for diagnostics of apicomplexan pathogen in ticks and mammalian hosts

**DOI:** 10.1186/1756-3305-7-S1-O13

**Published:** 2014-04-01

**Authors:** MA Qablan, F Boyer, C Miquel, G D'Amico, AD Mihalca, F Pompanon, D Modrý

**Affiliations:** 1 Department of Pathology and Parasitology, University of Veterinary and Pharmaceutical Sciences, Brno, Czech Republic; 2 CEITEC-Central European Institute of Technology, University of Veterinary and Pharmaceutical Sciences, 61242 Brno, Czech Republic; 3 Laboratoire d'Ecologie Alpine, Université Joseph Fourier, Grenoble, France; 4 Department of Parasitology and Parasitic Diseases, University of Agricultural Sciences and Veterinary Medicine, 400372 Cluj-Napoca, Romania

## 

Among apicomplexan parasites, ticks are known vector of several species belongs to three protozoan genera (*Babesia*, *Theileria *and *Hepatozoon*). During their life cycle, tick-transmitted apicomplexan parasites alternate between asexual (in vertebrate host) and sexual (in ticks) developmental stages. The major constraint for the proper diagnostics of those pathogens is the high possibility of mix infection, both in ticks and vertebrate hosts, with several species or genotypes. The aim of this study was to apply the Next Generation Sequencing (NGS) as a method of choice for simultaneous determination of the full spectrum of apicomplexan pathogens in ticks and the mammalian hosts. Therefore, A pair of universal primers were designed to flank a 167 bp barcode region of the 18s rRNA gene of all *Babesia*, *Theileria *and *Hepatozoon *species. The new protocol was evaluated on DNA samples isolated from 195 dogs and 144 ticks (*Rhipicephalus armatus *and *R. pulchellus*) collected from Northern Kenya. In total 301 sample (89%) were positive for apicomplexan infections; ranging from single to multiple infection with one species or several species and/or genotypes in a single sample. The most abundant apicomplexan pathogens were *Hepatozoon *followed by *Babesia *and *Theileria*, respectively. Further, the result shows that the barcode region entails enough variability that allows identifying the pathogens up to the subspecies and genotypes level. The exact methodological and results detailed will be presented later. This work was supported by the project OP VK CZ.1.07/2.3.00/30.0014.

